# Sexual satisfaction of postmenopausal women: An integrative review

**DOI:** 10.1371/journal.pone.0306207

**Published:** 2024-07-30

**Authors:** Nasim Shahrahmani, Raheleh Babazadeh, Abbas Ebadi

**Affiliations:** 1 Department of Midwifery and Reproductive Health, Student Research Committee, School of Nursing and Midwifery, Mashhad University of Medical Sciences, Mashhad, Iran; 2 Nursing and Midwifery Care Research Center, Mashhad University of Medical Sciences, Mashhad, Iran; 3 Department of Midwifery, School of Nursing and Midwifery, Mashhad University of Medical Sciences, Mashhad, Iran; 4 Behavioral Sciences Research Center, Life style institute, Baqiyatallah University of Medical Sciences, Tehran, Iran; 5 Nursing Faculty, Baqiyatallah University of Medical Sciences, Tehran, Iran; Universidade Estadual de Campinas, BRAZIL

## Abstract

A prerequisite for interventions for sexual satisfaction in postmenopausal women is a clear, objective measurement of the concept. Despite the large number of studies on the sexual satisfaction of postmenopausal women, there is no clear definition of sexual satisfaction in menopause. This study was conducted to investigate the concept of sexual satisfaction in postmenopausal women. The present study was carried out using an integrated review of data obtained from secondary sources, utilizing Whittemore and Knafl’s method of bibliographic search. A literature search was performed without any data limitations in journals and international databases. The primary inclusion criterion was relevance to sexual satisfaction in postmenopausal women. The full texts of all these articles were evaluated using the checklists of the MMAT and PRISMA. Data were analyzed using MAXQDA 10 software using a constant comparison method. Meaning units were identified and coded. The codes were classified into subgroups and categories according to the characteristics, antecedents, and consequences of sexual satisfaction in postmenopausal women. During the integrative review of the 62 articles and three books, 580 codes about sexual satisfaction in menopause were extracted. The codes were grouped into three main attributes, five main antecedents, and three main consequences of sexual satisfaction in postmenopausal women. Four attributes, symptoms, or components were identified for the concept. These attributes were as follows: Change in sexual objective and subjective dimensions of sexual satisfaction after menopause, conditional sexual consent, change in behavior, and sexual function. These dimensions distinguish sexual satisfaction in menopause from other conditions. The concept of sexual satisfaction in menopause is a subjective (emotional interaction) and objective (physical interaction) experience that is conditioned by the fulfillment of expectations and the reconstruction of sexual relations while also being influenced by the change in sexual capacity during menopause.

## Introduction

Menopause is a natural biological process that affects women’s lives physically, mentally and sexually [1]. The global population of postmenopausal women is growing. In 2021, women aged 50 and over accounted for 26% of all women and girls [2]. Approximately one-third of women’s lives are postmenopausal, and almost all studies show that the vast majority of women and men are in good health and continue to be sexually interested and active with a suitable partner throughout their lives [3].

In Addis (2006), examining the sexual activities and behaviors of 2109 women between the ages of 40 and 69, it was shown that a significant proportion, approximately 75%, remained engaged in sexual activity despite the typical decline in sexual performance associated with advancing age. [3]. Sexual satisfaction is a component of human sexuality that occurs at the end of the sexual response cycle [5]. Sexual satisfaction is one of the recognized sexual rights, and everyone has the right to a satisfying and enjoyable relationship [6].

According to Hudson (1981), sexual satisfaction is the degree of harmony and contentment with sex [4]. In developing the idea of interpersonal exchange, Lawrence and Bayer (1992) define sexual pleasure as an emotional response arising from a subjective evaluation of the positive and negative aspects of a person’s sexual relations [5]. Sex researchers are interested in sex life satisfaction for two reasons: first, sexual satisfaction provides a mechanism for assessing the performance of relationship partners [6]; second, it has been found that sexual satisfaction is a predictive factor for various relationship dimensions, including marital quality and stability [7, 8].

Estrogen, which is vital in sexual desire, is one of the influential variables in sexual satisfaction. Estrogen aids in the elasticity of pelvic tissues for sexual intercourse. Vaginal dryness can occur when estrogen is not produced sufficiently before menopause. In addition, other factors influencing sexual satisfaction are physical sexual performance, such as stability in orgasm or frequency or time of orgasm [9]. Furthermore, aging-related diseases reduce sexual desire, physiological response to the sexual cycle, and, as a result, sexual satisfaction [10, 11].

Menopausal women have a very different concept of sexual satisfaction than premenopausal women, and this difference is due to aging and physical, hormonal, cultural, and psychological changes. Examining the concept of sexual satisfaction from the perspective of menopausal women is critical for sexual education and sexual therapy because the type of education and the content of education are very different from those before menopause due to menopausal changes [12–14].

In general, there is little information and no consensus on the concept and definition of perceived sexual satisfaction in menopause [9–11].

According to Schwartz and Young (2009), the absence of a consensus definition and the existence of multiple definitions of the term "sexual satisfaction" have led researchers to conduct individual assessments rather than those based on feelings and behavior. According to Schwartz and Young, cases that separate specific elements from the psychological structure are relatively unknown in the literature on sexual satisfaction. Regardless of the truth of this issue and the possibility that sexual satisfaction research will continue in the same manner, a comparison of Schwartz and Young’s statements with other studies reveals an important point: researchers should not rely on the idea that satisfaction is inherently subjective and that everyone knows what it means [10, 11].

As McClelland (2010) pointed out, the study of sexual satisfaction is not complete and most research has been conducted using self-reinforcing definitions of sexual satisfaction to measure the construct of sexual satisfaction [15]. When researchers disagree on the meaning of a concept, conducting coherent research yields a clear definition [21]. When the goal is to create and generalize accurately about a phenomenon with little information, an integrative review can be used [22]. Accordingly, integrative review has been advocated as necessary for evidence-based practice initiatives in nursing [16, 17]. Several studies have been conducted regarding sexual satisfaction in menopause. However, since no study summarized all of these cases and presented them coherently, as well as a lack of a single and coherent definition of sexual satisfaction in menopause, the current integrated review study was conducted to come up with the concept of sexual satisfaction in menopause.

## Materials and methods

The study was carried out using an integrated review of data obtained from secondary sources, utilizing Whittemore and Knafl’s method of bibliographic search [17]. Conducting an integrative review, which examines the articles in the reference for similarities and differences, is one of the most effective ways to begin a study. A clearer and more comprehensive comprehension of the concept is achieved through the use of this approach [18]. The five steps of Whittemore and Knafl’s method are as follows: Problem identification stage, literature search, data evaluation, data analysis, and presentation [17].

### Stage 1. Problem identification stage

This method begins with the precise identification of the issue that requires investigation. The variables of interest (i.e., concepts, target population, health care problem) and the suitable sampling frame (i.e., type of empirical studies, inclusion of theoretical literature) subsequently establish the objective [17].

The concept of "sexual satisfaction of postmenopausal women" was taken into account. The original research questions were presented as described below: "How can the concept of sexual satisfaction in postmenopausal women be clearly defined? "How is the concept of sexual satisfaction defined by postmenopausal women? And "What are the uses of the concept of sexual satisfaction in postmenopausal women in the texts?"

### Stage 2. Literature search

A comprehensive search for an integrative review typically involves using a minimum of two to three strategies to identify the greatest number of eligible primary source [17, 19]. The investigation was carried out using the international databases as follows:

Google Scholar, CINAHL, Embase, Medline via OVID, Psych INFO, Web of Science, Cochrane, ScienceDirect, ProQuest, and Scopus. Search keywords were (“postmenopausal”, “menopause”, “postmenopausal women”, “aged”, “older adults”, “older women”, “Midlife”, “middle-aged”), and (“sex* satisfy*”, “satisfy* sex*”, “satisfaction with sex”, “sexual satisfaction”, “Dissatisfaction”) and (“concept”, “meaning”, “dimensions”, “predictors”, “affecting factors”). Keywords were combined using the Boolean operators “AND” and “OR”. This study had several search stages. After reviewing the articles, the following dimensions and concepts were searched for: “gender, socio-cultural factors, relational factors, positive emotions, sexual desire, mental factors, orgasm, sense of security, sexual performance, emotional intimacy, self-esteem, body image, dyspareunia, vaginal dryness, socioeconomic status, marital satisfaction, life satisfaction”.

**Fig 1:**
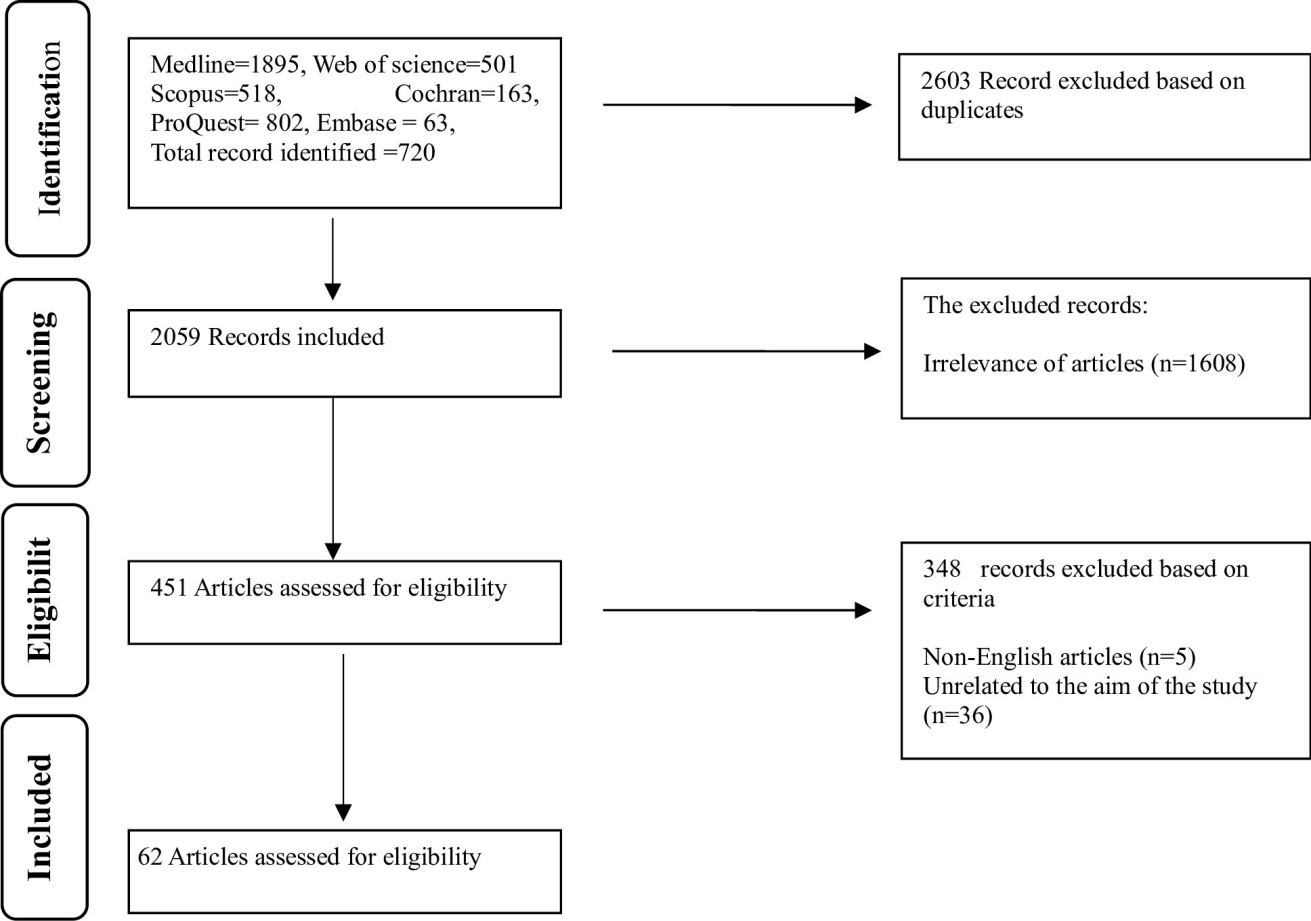
Shows the PRISMA flow diagram.

The integrated review literature search was conducted without regard to time limits until March 30, 2024. The literature search was carried out by the four-step preferred reporting for systematic reviews and meta-analyses PRISMA flow diagram [20], as shown in Fig 1.

In addition to conducting a manual search of Google Scholar and midwifery, psychology, and mental health textbooks were studied as well for articles about the concept of sexual satisfaction in postmenopausal women. Two independent researchers conducted the literature search and assessed the eligibility of the articles (the first author and the corresponding author). An assessment was conducted on the titles and abstracts of the acquired articles, and those that failed the eligibility criteria were omitted. Articles published in reputable academic journals and directly relevant to the goals and research questions met the inclusion criteria. The study’s exclusion criteria included articles or journals that were irrelevant, as well as short articles such as editor’s comments, suggested comments, abstracts, and articles presented at conferences. We used the articles in international databases without any language-based limitations.

Articles completely related to the purpose of the concept analysis stage are selected in the final evaluation stage of the articles by preparing questions about the purpose of the research.

The initial literature search yielded 4662 records, of which 2603 were deemed unnecessary due to being duplicates. The software End Note was utilized to identify and eliminate duplicate records. The titles and abstracts of the remaining articles were assessed during the screening phase, during which 1608 articles were deemed irrelevant to the study’s objectives and inquiries and were therefore excluded. 451 articles were also omitted on account of their ineligibility. In the end, 62 articles were incorporated into the research. The Persian language articles in Scopus and Cochrane were retained, and a Chinese article with an English summary was translated and included in the research. The literature search yielded three textbooks relevant to the study’s objectives and inquiries.

### Stage 3. Data evaluation

The 62 articles included in the integrative review were clinical trials, reviews, descriptive studies, cohort, systematic reviews, qualitative studies, a cross-sectional and three books. Table 1 shows the characteristics of the included studies. Classification of articles was done based on timing. The full-texts of all these articles were assessed using the checklists of the Mixed Methods Appraisal Tool (MMAT) 2018 and the MMAT is a critical appraisal tool designed for the appraisal stage of systematic mixed studies reviews, i.e. reviews that include qualitative, quantitative, and mixed methods studies. It permits to appraise the methodological quality of five categories of studies: qualitative research, randomized controlled trials, non-randomized studies, quantitative descriptive studies, and mixed methods studies [21].

**Table 1:** Characteristics of included studies.

No.	Authors/Year/Country	Aims	Study design	definition
1	Walfisch/1984/North African Jewish origin	Sexual satisfaction among middle-aged couples and correlation with frequency of intercourse and health status [23].	Correlation	Sexual satisfaction is defined more by their emotional relationship with their husbands than by the physical aspects of sex.
2	jobes/1986/Caucasian	Examines sexual experiences and satisfaction among a homogeneous sample of middle-aged [24].	A cross-sectional	Sexual Satisfaction is implicitly defined by voluntary association memberships serendipitously emerged as a relatively strong indicator of social activities related to sexual satisfaction.
3	Davidson/1995/United States	The purpose of this was to ascertain the relationship, if any, between the sexual behaviors, religiosity, and sexual satisfaction of women [9].	A cross-sectional	Sexual satisfaction is implicitly defined by religious balance.
4	Young/2000/United States	Evaluation of the relationship between marital satisfaction, orgasm, frequency of sexual activity, and noncoital Sexual Activity with sexual satisfaction [25].	Correlation	Sexual satisfaction was "satisfaction with nonsexual aspects of the relationship".
5	Park/2003/Korea	Examine relationships between the sexuality and demographic, body massindex, and life style factors including smoking, alcohol use, and physical activity [26].	Cross-sectional	Sexual satisfaction is an important aspect of the quality of life or well-being of postmenopausal women.
6	Hartmann/2004/Germany	The purpose of this was to assess differences in determinants of sexual satisfaction, present sexuality, and personality factors between younger and older women [27].	A series of studies	shift in the individual experience and concept of sexual satisfaction with growing age that enables a woman to retain or even strengthen her sexual satisfaction.
7	Yeh/2006/Iowa	Evaluation of the Sexual satisfaction, marital quality, and marital instability [28].	Cross-lagged models	Sexual satisfaction is implicitly defined by the quality of marital relations.
8	Dennerstein/2006/Italy	To determine the relationship between low sexual desire and sexual or partner relationship satisfaction [29].	Cross-sectional	Sexual satisfaction is implicitly defined by the presence of desire.
9	Davison/2008/Australian	Describe sexual function and sexual satisfaction pre(PreM) and postmenopausal (PM) women [8].	Cross-sectional questionnaire	Sexual Satisfaction is implicitly defined by reported more sexual thoughts, interests, events, and initiation of sexual activity.
10	McCall-Hosenfeld/2008/United States	Describe factors associated with sexual satisfaction among sexually active postmenopausal women [30].	Cross-sectional analysis.	Satisfaction with sexual activity among postmenopausal women is implicitly defined by demographic and historical factors that are not amenable to physician intervention.
11	McCall-Hosenfeld/2008/United States	The objectives of this study were to explore the association of sexual satisfaction with prevalent cardiovascular disease and cardiovascular disease risk factors among sexually active postmenopausal women [31].	Observational cohort	Sexual satisfaction is implicitly defined by a lack of psychosocial stress and organic pathology.
12	Penhollow/2009/Florida	Investigate the impact of sociological, cultural, and psychological factors to further explain variance beyond biological changes that influence participation in sexual intercourse, sexual satisfaction, and overall quality of life [32].	Cross-sectional analysis	Sexual satisfaction is implicitly defined by sexual self-confidence, frequency of orgasm, and relationship satisfaction.
13	Dundon/2009/Burlington, VT, USA	This study aims to explore the empirical evidence regarding factors that predict sexual satisfaction in middle-aged women [33].	Cross-sectional	Sexual satisfaction is implicitly defined by psychological well-being, relationship adjustment, and, to a lesser extent, menopausal symptoms and sexual function.
14	Carpenter/2009/Chicago	This article explored the social factors contributing to Sexual Satisfaction differences [34].	Cross-sectional	Sexual satisfaction has two dimensions: physical and emotional. Women’s emotional satisfaction was closely associated with bodily sexual practices.
15	Davison/2009/Australia	To explore the relationship between self-perceived satisfaction and well-being with sexual function in women and to determine if there is an independent effect of menopausal status or age [35].	Cross-sectional	Dissatisfaction includes lower psychological general well-being.
16	Eftekhar/2009/Iran	The aim of this study was objectives to clarify the effect extended by Hormon Replacement Therapy (HRT) in improving post menopausal mood disturbances [36].	Clinical trial	Sexual satisfaction is implicitly defined by improved menopausal- related mood and sleep disturbances.
17	Pazandeh/2010/Iran	This study was conducted to determine the affecting agents on the sexual satisfaction of postmenopausal [37].	Descriptive study	Sexual satisfaction is implicitly defined by education level, leading to increasing knowledge and adaptation with menopause.
18	Woloski-Wruble/2010/Hebrew literacy	To investigate the sexual activities of older women and their levels of life satisfaction, sexual satisfaction and to examine the relationship between the level of sexual activities, sexual satisfaction, and life satisfaction [38].	A descriptive, correlational study	Sexual satisfaction is defined as life satisfaction.
19	Heiman/2011/Brazil, Germany, Japan, Spain, and the U.S	Examine the sexual and relationship parameters of middle-aged couples in committed relationships lasting 1 to 51 years [39].	Survey research	Sexual satisfaction plays an important role in physical intimacy in a long-term committed relationship.
20	Thompson/2011/San Diego	To determine if measures of successful aging are associated with sexual activity, satisfaction, and function in older postmenopausal women [40].	Cross-sectional study	Sexual satisfaction is implicitly defined by perceived quality of life, successful aging, and Subjective assessments appear to be stable in the midst of age-associated changes in physical, mental, and sexual health.
21	Trompeter/2012/California	describe the prevalence and covariates of recent sexual activity and satisfaction in older community-dwelling women [41].	Descriptive	Sexual satisfaction is implicitly defined as achieved sexual satisfaction through touching, caressing, or other intimacies that have developed over the course of a long relationship.
22	Kim/2012/Korean	To identify gender-related factors influencing sexual satisfaction [42].	Cross-sectional survey	Sexual satisfaction is implicitly defined by achieved marital satisfaction, followed by frequency of sexual activity, absence of depressive symptoms, age, and length of cohabitation with spouse.
23	Ornat/2013/Spanish	To assess sexual function, satisfaction with life (SWL), and menopause-related symptoms among mid-aged [43].	Cross-sectional study	Sexual satisfaction is a general sense of well-being that is dependent on expectations being met.
24	Fisher/2015/Japan, Brazil, Germany, Spain, and the United States	The aim of this study was to reports a dyadic analysis of sexual satisfaction, relationship happiness, and correlates of these couples [44].	Cross-sectional	Sexual satisfaction is implicitly defined by relationship happiness always depended on identifying and understanding mutual.
25	Holly/2015/US	The aim of this study was to give updated prevalence estimates of sexual activity among women and to elucidate factors associated with sexual activity and sexual satisfaction [45].	Cross-sectional	Sexual satisfaction is implicitly defined by Psychosocial factors (relationship satisfaction, communication with romantic partner, and importance of sex).
26	Smith, BS/2015/Washington	The aim of this study was what factors contribute to sexual satisfaction in Middle-aged adults individuals [46].	Cross-sectional survey	Sexual satisfaction is defined by sexual function.
27	Najar/2015/Iran	The present study was conducted with the aim of investigating the effect of fennel vaginal cream on painful intercourse and sexual satisfaction of postmenopausal women [47].	Clinical trial	Sexual satisfaction is implicitly defined by the reduction of dyspareunia.
28	Yang/2016/Taiwanese	The aim of this study to examine Taiwanese women’s perspectives on the way menopause affected their sexual behavior to gain an in-depth understanding of their experiences during this transition [48].	A qualitative	Sexual satisfaction is defined by the sociocultural view that women’s role is to satisfy their partner’s sexual appetite.
29	Kavlak/2017/Ankara	This study was conducted to determine the level of anxiety of postmenopausal women on their sexual satisfaction [1].	Descriptive study	Sexual satisfaction is defined as satisfaction and absence of anxiety.
30	Abedi/2017/Iran	The aim of this study was to examine education for sexual satisfaction [49].	Clinical trial	Sexual satisfaction is defined as improving awareness through group training.
31	Yosefzadeh/2017/Iran	The effect of date palm pollen capsule on sexual satisfaction and orgasm in menopausal women [50].	Clinical trial	Sexual satisfaction is defined by the improvement of orgasm.
32	Postigo/2017/ Brazil	To study the effects of Tribulus terrestris on sexuality in post-menopausal women [51].	Clinical trial	Sexual satisfaction is implicitly defined by the improvement of sexual performance.
33	Štulhofer/2017/Norway, Denmark, Belgium and Portugal	The aim of the present study was to explore the role of sexuality in successful aging [52].	Three-dimensional model of SA and examined its associations	Sexual satisfaction is implicitly defined by successful aging (SA).
34	Mazalzadeh/2018/Iran	The aim of the present study was to assess the effect of fenugreek vaginal cream on sexual satisfaction and dyspareunia in menopausal women [53].	Clinical trial	Sexual satisfaction is implicitly defined by the reduction of dyspareunia.
35	Afshari/2018/Iran	Determine the factors affecting sexual function and satisfaction in middle-aged women [54].	Cross-sectional	Sexual satisfaction is defined by adequate sexual function scores.
36	Beyazit/2018/Turkey	The study aimed to determine the association of menopausal characteristics with sexual satisfaction and marital adjustment [55].	Cross-sectional	Sexual satisfaction is defined by marital satisfaction.
37	Séjourné/2018/France	This study aimed to examine how the menopausal transition influences body image and satisfaction with sexual life [56].	Correlation	Sexual satisfaction is defined by positive body image.
38	Babayan/2018/Iran	The study aimed to determine the association of body image, sexual satisfaction and marital adjustment in middle-aged married women [57].	Correlational	Sexual satisfaction is implicitly defined by body image, marital adjustment, demographic and health factors.
39	Tadayon/2018/Iran	This study aimed to investigate the effect of Tribulus terrestris on sexual satisfaction in postmenopausal women [58].	Double‑blind clinical trial	The improvement of sexual desire and performance with the Tribulus Terrestris plant is defined as sexual satisfaction.
40	Davis/2018/USA	To investigate the effects of IVT on sexual satisfaction, vaginal symptoms, and urinary incontinence (UI) associated with AI use [59].	Double-blind, randomized, placebo-controlled trial	Sexual satisfaction is implicitly defined by a reduction in painful sexual intercourse.
41	Ansari/2019/Iran	The study was conducted to investigate the effect of group counseling with an approach to emotional regulation on the sexual satisfaction of postmenopausal women [60].	Double‑blind clinical trial	Sexual satisfaction is achieved when the couple’s emotional self-disclosure and awareness are established.
42	Niazi/2019/Iran	The purpose of this study was to investigate the effect of medicinal plants on the sexual satisfaction and function of postmenopausal women [61].	A Review	Sexual satisfaction is implicitly defined by the improvement of dyspareunia and sexual function. Medicinal herbs containing phytoestrogens can be considered as a selective treatment for the improvement.
43	Thomas/2019/Pittsburgh	The purpose of this study was to find out how body image affects sexual performance and satisfaction in middle-aged women [62].	A qualitative	Sexual satisfaction is implicitly defined by positive body image.
44	Movahed /2019/Iran	The main goal of this study was to examine some demographic and sociocultural factors affecting sexual satisfaction among postmenopausal women [63].	Cross-sectional	Sexual satisfaction is implicitly defined by the use of strategies to improve Sexual satisfaction by lifestyle, and sexual performance.
45	Kelley/2019/United States	To investigate associations between past-year verbal, and physical abuse and sexual (dis)satisfaction among postmenopausal women [64].	A cross-sectional analysis	Women’s sexual satisfaction is a biopsychosocial model, not just biological.
46	Traen/2019/Norway, Denmark, Belgium, and Portugal	This article aimed to describe partnered and non partnered sexual satisfaction and sexual activity in older men and women [65].	Correlational	Sexual satisfaction is implicitly defined as having access a permanent partner.
47	Blumenstock/2019/Canada	The purpose of this study was to examine highly satisfying relationships from less satisfying relationships in middle age [66].	Relationship descriptive	Sexual satisfaction is implicitly defined by frequent partnered sexual activity that predicts high emotional satisfaction.
48	Harder/2019/UK	The study aimed to examine sexual activity, functioning, and satisfaction in a large sample of postmenopausal women [67].	A qualitative	Sexual satisfaction is implicitly defined as having an intimate partner and good physical health.
49	Abedi/2020/Iran	This study was conducted to investigate the relationship of empty nest syndrome on sexual satisfaction [68].	Cross-sectional	Sexual satisfaction is influenced by psychological factors.
50	Bosak/2020/Iran	this study was to determine the effect of chamomile vaginal gel on sexual satisfaction and dyspareunia in postmenopausal women [69].	Clinical trial	Sexual satisfaction is implicitly defined by a reduction in painful sexual intercourse.
51	Buczak-Stec/2020/German	This study was to investigate the determinants of sexual satisfaction longitudinally among middle-aged and older adults [70].	Longitudinal study	Sexual satisfaction is implicitly defined by lower number of physical illnesses, better self-rated health, absence of depression and higher importance of sexuality and intimacy.
52	Arenella/2020/United States	This study examined an ecological model of sexual satisfaction in midlife women in relationships, and paid particular attention to the role of intergenerational caregiving in predicting satisfaction [71].	Ecological model	Sexual satisfaction is implicitly defined by with income, affectual solidarity, and sexual function.
53	Thomas/2020/ Pittsburgh	This study was to explore older women’s perceptions of causes of low libido [72].	A qualitative	Sexual satisfaction is implicitly defined by biological, interpersonal, social, and psychological factors
54	Karimi/2021/Iran	The study aimed to investigate the effect of squill oil on dyspareunia and sexual satisfaction in menopausal women [73].	Clinical trial	Sexual satisfaction is implicitly defined by a reduction in painful sexual intercourse.
55	Hajihasani/2021/Iran	the present study aimed to investigate the predictive role of demographic and psychological factors affecting sexual satisfaction in women after 40 years [74].	Cross-sectional	Sexual satisfaction is implicitly defined by Having psychological factors (i.e., mindfulness and body image).
56	Leonhardt/2021/USA	the present study aimed to assess what trajectories of sexual satisfaction exist in midlife marriages [75].	Longitudinal study	Sexual satisfaction is implicitly defined by marital satisfaction and marital stability.
57	Hashemzadeh/2022/Iran	This study aimed to evaluate the effect of cardiac rehabilitation after coronary artery surgery in postmenopausal women on their sexual satisfaction [76].	Cross-sectional	Improvement of sexual satisfaction with previous surgical rehabilitation program after coronary artery surgery in postmenopausal women.
58	Elbiss/2022/ Emirates	Conducted a systematic review among patients undergoing treatment with DQRF for genitourinary atrophy and improvement in sexual Satisfaction [77].	Systematic review	Sexual satisfaction is implicitly defined by the improvement of vaginal atrophy.
59	Khazaeian/2023/Iran	The study sought to evaluate the effects of mindfulness-based counseling on sexual self-efficacy and sexual satisfaction among Iranian postmenopausal women [78].	Quasi-experimental	Sexual satisfaction is implicitly defined by improved sexual self-efficacy.
60	Mutz, Rosa/2023/ Dutch	The present study aimed to further understand this mechanism by investigating the mediating role of sexual assertiveness in the relationship between sexual self-esteem and Sexual satisfaction in men and women [79].	Cross-sectional design	Sexual satisfaction is implicitly defined by sexual self-esteem.
61	Khakkar/2023/ Iran	This study evaluated the relationship between depression and sexual satisfaction in middle-aged women by evaluating the equation model analysis of the two models [80].	Cross-sectional	Sexual satisfaction is implicitly defined as the absence of anxiety in sexual relations and the absence of depression.
62	Wang/2023/ China	aimed to investigate the prevalence and correlates of sexual activity and sexual satisfaction among older adults in China [81].	Cross-sectional	Physical satisfaction is defined as better general health without disability.
63	Edited ByStephen B. Levine First Published 2011	Handbook of Clinical Sexuality for Mental Health Professionals [82].	BOOK	Sexual satisfaction is implicitly defined by a more relaxed and emotionally skilled relationship.
64	Emily An Impett Chapter January2012	Because it feels good: Toward a positive psychology of sexuality [83].	BOOK	Sexual satisfaction is implicitly defined by frequent joint sexual activity.
65	American Psychiatric Association 2013	Diagnostic and Statistical Manual of Mental Disorders, 5th Edition (DSM-5) [84].	BOOK	Sexual satisfaction is implicitly defined by a reduction in painful sexual intercourse.

The PRISMA 2020 Checklist was utilized for the evaluation of articles. The PRISMA checklist comprises 27 items that pertain to the content of a systematic review and meta-analysis. It encompasses sections such as abstract, methods, results, discussion, and financial resources [22]. The scoring method for this checklist is as follows: each item mentioned in the articles receives a score of one, while items not mentioned receive a score of zero. The score range is from 0 to 20. Articles with a low score (less than 10) were excluded from the study using this checklist. During the final evaluation stage, articles that are directly relevant to the purpose of the concept analysis stage are chosen. The following questions were also used for eligibility assessment: “Do the articles describe and define sexual satisfaction in postmenopausal women?”, “Do these articles address the characteristics of sexual satisfaction in menopausal women?”

No studies were excluded at this stage.

### Stage 4. Data analysis

In research studies, data analysis necessitates the sorting, coding, categorizing, and summarizing of information derived from primary sources. Its purpose is to arrive at a unified and cohesive conclusion regarding the research problem and to present the data in a comprehensive and unbiased manner. Integrated surveys utilize the same analysis strategies and data analysis. The four steps of this method are data reduction, data display, data comparison, and conclusion drawing and verification [17, 85].

For data management, the initial and subsequent phases, which included data reduction and data presentation, implemented a general classification system. Before classifying the data according to the time of publication in Table 1, the data were evaluated according to the type of evidence to facilitate the analysis of the integrated review. By examining the extracted data case by case, units were taken into account and systematically coded. The codes were shown in Table 2. Table 2 shows the categories and subcategories of the antecedents, attributes, and consequences of the concept of sexual satisfaction in menopause. Codes were compared repeatedly, and similar codes were assigned to subcategories and then main categories. The results were divided into the major categories of definitions, characteristics, antecedents, and consequences in the fourth step. MAXQDA 10 software was used for data management [17, 86].

**Table 2:** The categories and subcategories of the antecedents, attributes and consequences of the concept of sexual satisfaction in menopause.

Components	Categories	Subcategories
**Antecedents**	Individual and background characteristics	Demographic characteristics—economic and social characteristics—receiving health services—physical health—lifestyle—quality of sexual performance before menopause
	Interpersonal interaction pattern	Committed sex—quality of life with partner- physical and sexual violence—social support
	Variables related to spouse	Sexual disorder in the spouse—Insufficient sexual literacy—sexual coercion before menopause
	Religious and cultural values and beliefs	Cultural factors—adherence to religious beliefs
	psychological factors	Mental health problems—mental illnesses—a lack of sexual self-efficacy
**attributes**	Change in sexual capacity	Menopause-related unpleasant physical changes—menopause-related perceived psychological harm -
	Objective and subjective dimensions of sexual satisfaction after menopause	Emotional interaction of couples—physical interaction of couples
	Conditional sexual consent	Assessment/evaluation—the degree of fulfillment of expectations
	Changes in sexual behavior and performance	Sex beyond intercourse—seeking treatment in menopause
**Consequences**	Improving physical state	Health-seeking behaviors—physical health
	Improving the state of mental health	Positive attitude toward menopause—psychological well-being
	Promotion of sexual intimacy between couples	Emotional relationships—physical relationships

## Results

During the integrative review of the 62 articles, 3 books and 580 codes about Sexual satisfaction in menopause were extracted (Table 1). The results showed that there were several, non-inclusive definitions of sexual satisfaction during menopause. To give a better understanding of the concept, its attributes, antecedents, and consequences were extracted (Table 2). Through the analysis of the explicit and the implicit definitions sexual satisfaction during menopause five antecedents were identified for the concept.

### The antecedents of sexual satisfaction in menopause

Data analysis showed that the antecedents of the concept of sexual satisfaction in menopause are Individual and background characteristics [26, 31, 33–35, 37, 42, 43, 54, 70, 71], patterns of interpersonal interactions [24, 28, 34, 44, 46, 47, 63, 64, 71], variables related to spouse [25, 34, 42, 43, 52, 53, 55, 60, 63, 72, 80], religious and cultural values and beliefs [9, 24, 30, 52, 55], and psychological factors [1, 24, 34, 38], that are explained in the following.

### Individual and background characteristics

Individual and background characteristics [33, 70], age [33, 70], education level [54], income level [74, 87], financial independence [39], employment status [43], location of residence (in terms of social class) [70] and background variables such as receiving health care services [26], awareness and sexual wisdom [37, 71] are associated with sexual satisfaction. Based on this, older menopausal women, those with a lower level of education and awareness, those who receive less care services, and those with lower financial status and financial insecurity report less sexual satisfaction. In addition, physical health factors, such as diseases and drug use, have a negative impact on sexual satisfaction. In fact, healthy aging and the absence of sleep disorders enhance sexual satisfaction [26, 31, 34–36, 38, 45]. Menopausal women’s lifestyles, including smoking, effectively decrease sexual pleasure, while exercise and physical fitness help increase sexual satisfaction [26, 40, 56]. Furthermore, the quality of sexual performance before menopause and typical sexual performance are critical determinants of menopausal sexual satisfaction [32, 33, 42, 43, 46].

### Interpersonal interaction pattern

Long-term committed sex [46], a sense of safety in sex [64], or the possibility of marriage [83] can all contribute to an increase in sexual desire during menopause. Sexual satisfaction in menopause is positively impacted by the quality of a couple’s life before menopause, including marital satisfaction [44, 63, 75], life satisfaction [55], positive emotional connections between partners, satisfaction with nonsexual aspects of marriage [28], and the presence of love [34, 44]. Sexual satisfaction is negatively impacted by factors such as verbal and physical abuse [55, 64]. On the other hand, social support [71] and couples’ engagement in social activities [24] favorably impact sexual satisfaction in postmenopausal women.

### Variables related to spouse

Sexual disorders in the spouse, such as erection disorders [43] and ejaculation [63], as well as a decline in sexual desire in men as they age [34, 65], are factors in decreasing sexual satisfaction and physical and mental health [70, 72]. Inadequate sexual literacy of the spouse, such as a low level of education [1, 55], insufficient information on sex and menopause [48], and sexual unawareness of the couple [60], are among the factors that diminish the sexual satisfaction of postmenopausal women. Sexual coercion prior to menopause, which occurs due to the preservation of cohabitation [63], fear of betrayal by the spouse [42] or obtaining the sexual consent of the spouse [67] or forced marriage [42, 63] are among the influencing factors that have a negative impact on sexual satisfaction.

### Religious and cultural values and beliefs

Cultural factors play an important role in the formation of postmenopausal women’s values, attitudes, and beliefs [9], which can have a significant impact on sexual satisfaction. Traditional beliefs, cultural taboos, and cultural restrictions [55, 65] in the expression of sexual desires can all have an impact on sexual satisfaction, either positively or negatively. Couples of the same race have higher levels of sexual satisfaction [30]. Adherence to religious beliefs and any society’s teachings can have positive and negative effects on sexual satisfaction [9, 24].

### Psychological factors

Damaged mental health, such as guilt following a spouse’s death [67], feelings of depression and anxiety [1, 33], sadness [1, 33], thoughts of separation and failure [44, 87], thoughts of sexual abuse [33], feelings of fear and despair [24], feelings of loneliness and empty nest syndrome after children leave [24, 68] are among the factors that lower sexual satisfaction. Mental illnesses and bipolar disorders, as well as the use of psychoactive drugs, can all contribute to decreased sexual satisfaction [33]. Lack of sexual self-efficacy, including sex hatred and doubt [44, 87] and a lack of sexual thoughts and passivity [38], are also factors influencing sexual satisfaction in menopause.

### The attributes of sexual satisfaction in menopause

The attributes of sexual satisfaction in menopause through the analysis of the explicit and implicit definitions of sexual satisfaction in menopause four attributes, symptoms, or components were identified for the concept. These attributes were as follows: Change in sexual capacity [1, 23, 35, 39, 40, 50, 55, 57, 62, 67, 72], objective and subjective dimensions of sexual satisfaction after menopause [23, 27, 33, 55, 60, 66, 67, 78, 87], conditional sexual consent [33, 43, 55, 63, 73, 82, 87], change in behavior and sexual function [39, 45, 67].

### Change in sexual capacity

Sexual satisfaction in menopause decreases due to unpleasant physical events that occur due to hormonal and physical changes, such as painful intercourse, vaginal dryness, decreased vaginal flexibility, decreased libido, decreased occurrence of orgasms, decreased arousal, and menopausal symptoms [1, 23, 35, 39, 40, 50, 55, 57, 62, 67, 70, 72]. In addition to these perceived psychological effects of menopause, such as feeling sorry for not having sexual desire, feeling depressed and embarrassed after menopause, lack of self-confidence due to physical changes, unfavorable body image, and a sense of reduced attractiveness are also among the factors contributing to decreased sexual satisfaction in women. Women who have a positive body image, adequate information in this field, and have accepted the menopause process have higher levels of sexual satisfaction [1, 29, 57, 62].

### Objective and subjective dimensions of sexual satisfaction after menopause

The subjective dimensions of sexual satisfaction are actually the emotional interaction of sexual satisfaction couples that include happiness from sex, a pleasant feeling from the relationship and the value of the relationship for women, the absence of negative emotions, sharing sexual preferences, satisfaction from previous positive emotions, and a sense of comfort [27, 33, 55, 82]. The objective dimension of sexual satisfaction is the physical interaction of couples. The physical Interaction of sexual satisfaction includes the frequency of sexual intercourse, the frequency of orgasm, the number of satisfactory relationships, being sexually active, and the absence of dyspareunia [65–67, 78, 87].

### Conditional sexual consent

Between sex and evaluation, there is mental balance. In fact, a person’s subjective evaluation includes positive and negative dimensions related to sex, level of satisfaction, and marital satisfaction after menopause [33, 43, 55, 63, 73, 82, 87]. The degree of expectation fulfillment is a conscious cognitive assessment of the degree of expectation fulfillment, the agreement of desires with reality, the amount of money couples spend in their relationship, and the solution they arrive at. Additionally, there is a direct link between sexual satisfaction and the positive rewards of interactive experiences [33, 43, 55, 63, 73, 82, 87].

### Change in behavior and sexual function

Women adjust their behavior during menopause to cope with behavioral changes. By broadening the definition of sexual satisfaction beyond intercourse, sex can be used to express mutual relationships. In fact, having non-genital contact and not focusing solely on the sexual relationship may be important for experiencing sexual satisfaction. On the other hand, sexual satisfaction is significantly influenced by the perception of intimacy or emotional closeness, rather than the emphasis on physical actions [25, 40, 42], seeking treatment during menopause [36, 50, 53, 58, 61, 69, 73] is one of the behavioral changes in women. Women who experience vaginal dryness may use medical aids such as vaginal lubricants or herbal medicines to treat or alleviate menopausal symptoms [39, 45, 67].

### The consequences of sexual satisfaction in menopause

Consequences are events that occur as a result of the concept’s occurrence; in other words, they are considered the concept’s outputs. Three significant consequences were extracted in this regard: improvement of physical condition [49, 58, 69, 73, 81], improvement of mental health condition [46, 78, 79, 82], and promotion of sexual intimacy between couples [35, 51, 66, 87].

### The definition of sexual satisfaction in menopause

Antecedents of sexual satisfaction in menopause are a broad self-evaluation of external, internal, and interactive factors. A collection of perceptions, beliefs, and behaviors are heavily influenced by underlying factors (individual and contextual variables, interpersonal interaction patterns, spouse-related variables, psychological variables, cultural and religious values, and beliefs). In this regard, the concept of sexual satisfaction in menopause is a subjective (emotional interaction) and objective (physical interaction) concept that is conditioned by the fulfillment of expectations and the reconstruction of sexual relations, while also being influenced by the change in sexual capacity during menopause. As a result, the couple’s physical health improves, as does their mental health and sexual intimacy, allowing the marital relationship to continue satisfactorily.

## Discussion

An integrative review is a specific method that summarizes past empirical or theoretical literature to provide a more comprehensive understanding of a particular phenomenon or healthcare problem. This integrated study aims to examine sexual satisfaction in postmenopausal women and identify its antecedents, characteristics, and unique consequences during this time in women’s lives.

Antecedents are factors that must have existed before the concept’s occurrence. The antecedents helped the researcher understand the background and social context of the used concept, resulting in its refinement [88]. This study evaluated the antecedents of sexual satisfaction in menopause on a broad scale, encompassing external, internal, and interactive factors, a collection of perceptions, beliefs, and behaviors that are heavily influenced by underlying factors (individual and contextual variables, patterns of interpersonal interactions, variables related to spouses, psychological variables, cultural and religious values, and beliefs) that influence sexual satisfaction in menopause.

According to Walfisch et al.’s (1984) also discovered that various background factors influence women’s sexual satisfaction [23]. Age is an important factor in predicting women’s sexual satisfaction, and most research indicates that aging-related variables reduce sexual satisfaction [70, 89, 90]. Byers and Rahman (2014) discovered in a review that the age-related decline in sexual satisfaction is caused by other demographic or sexual variables associated with older age, such as partner habituation and health status [89]. According to Trompeter’s study, nearly half (47.5%) of women over 80 reported sexual satisfaction almost always. In this study, sexual activity was not always required for sexual satisfaction. Those who were not sexually active may have found sexual satisfaction through long-term intimacy, such as touching or caressing [41].

The research findings indicate that economic and social factors are additional underlying factors that impact the concept of sexual satisfaction. Among older women, factors such as residence are important for sexual satisfaction [70]. Good economic status can significantly increase sexual performance and sexual satisfaction [43]. Women who work have more self-confidence because they are financially independent and have more sexual desire than housewives, which affects sexual satisfaction. The sexual arousal of postmenopausal women and their husbands increases as their education level rises, which can lead to an increase in sexual satisfaction [37]. However, it should be noted that as couples’ educational status rises, they may have higher expectations for sexual satisfaction, and when these expectations are not met, they may experience sexual dissatisfaction [55].

Awareness of the menopause process can cause changes in attitudes and thoughts about sex, reduce anxiety, and assist a person in adapting to menopause while increasing sexual satisfaction [37].

Postmenopausal women who are supported and educated by health care providers are more sexually satisfied. which was consistent with the research results of Abedi et al. (2017) and Kurlychek et al. (1979) [14, 49]. Furthermore, a cause-and-effect relationship exists between sexual satisfaction and lifestyle factors. The study by Movahed et al. (2019) demonstrated that a person’s attitude toward menopause, sexual activity, and sexual satisfaction is influenced by their lifestyle. Therefore, women who live a healthy lifestyle with regular physical activity will have more sexual satisfaction [63], while age-related diseases, sexual desire, and the physiological response of the sexual cycle will reduce sexual satisfaction [34]. Insomnia, for example, has been shown to reduce women’s sexual desire and satisfaction [26]. Physical well-being is defined as a low risk of illness and disability, mental well-being as high cognitive and physical functional capacity, and emotional and social well-being as active engagement with life, all of which have a direct positive relationship with sexual satisfaction [38], which is consistent with the findings of our study.

Our research findings align with the notion that the occurrence and frequency of orgasm before menopause are significant indicators of sexual satisfaction following menopause. Heiman et al.’s (2011) study in five countries showed that the level of sexual performance before menopause significantly influences sexual satisfaction [39].

The pattern of interpersonal interactions is one of the factors influencing sexual satisfaction, with the assumption that women invest more in more committed relationship [34]. People check, determine, and judge their level of sexual satisfaction based on marital satisfaction. It is possible to argue that primary marital satisfaction is necessary for a satisfying benefit from sexual relations with a spouse, particularly for women [91]. In menopause, the relationship between sexual satisfaction and marital satisfaction is a two-way street; that is, marital satisfaction predicts subsequent sexual satisfaction, and satisfaction with sexual satisfaction is associated with increased marital satisfaction. People usually base their level of sexual satisfaction on marital satisfaction, and love between couples increases sexual satisfaction [34, 44], which is consistent with the findings of our study.

Another finding of this study was the effect of violence and social support on sexual satisfaction during menopause. However, low relationship satisfaction can be viewed as both a cause and an effect of violence. The problem of domestic violence among couples worsens with age. Intimacy and sexual satisfaction between spouses are destroyed by sexual and nonsexual violence [55, 92]. When women feel more supported, they have higher sexual satisfaction [93]. Social support is defined as a person’s evaluation of support and feeling of belonging [43]. When emotional self-disclosure and awareness of each other are established, sexual satisfaction is achieved [60]. Lack of knowledge about the wife’s menopause and sexual issues impacts sexual satisfaction [48].

The results of our research showed that men’s sexual dysfunction causes dissatisfaction in menopausal women. Researchers have shown that erectile dysfunction leads to dissatisfaction in women [67, 91].

The findings of this study revealed that culture has a significant impact on sexual satisfaction in postmenopausal women. Parke (1991) demonstrated that culture influences attitudes and beliefs about sexuality and sexual relationships. As a result, cultural beliefs can sometimes interfere with normal sexual function and cause harm, so addressing this issue is critical and necessary [94]. Cultural values and norms also influence sexual expression and satisfaction. For instance, in religious and patriarchal cultures like Asian societies, expressing sexual desires, especially for women, is considered inappropriate and a threat to the integrity of the family and society, leading to low sexual satisfaction [34]. Based on this, culture has a significant impact on gender and sexual satisfaction or dissatisfaction [63], which is consistent with the findings of our study.

In a review by del Mar Sánchez-Fuentes et al. (2014) the effect of religion on sexual satisfaction varied between studies. For example, some researchers discovered that higher religious beliefs were associated with lower satisfaction in white participants, whereas others discovered no discernible differences in sexual satisfaction by religion [90]. It has also been discovered that having a non-religious childhood correlates with higher sexual satisfaction in women [95]. However, women who attend church often report greater sexual satisfaction [24].

The findings of our study revealed that psychological factors are among the antecedents influencing sexual satisfaction. The transition from the fertile to the infertile period is associated with a variety of physical and psychological symptoms, and women go through a stressful period that can affect all aspects of their lives. According to Dundon and Rellini (2010), in women over the age of 40, mental health has a more significant impact on sexual satisfaction than physical performance [33], and depression, anxiety, and stress problems are all linked to lower sexual satisfaction [89, 90]. Yangın et al. (2008) discovered a negative and significant relationship between loneliness and all sexual components [96]. Sadness, fear, thoughts of failure and separation, frustration, and injury are all significant predictors of sexual dissatisfaction [80].

However, not all studies agree on the exact link between depression and sexual satisfaction. One study found a link between depressive symptoms and lower sexual satisfaction [97].

Concepts are identified by their most frequent occurrences in the texts; defining these characteristics gives the concept the most comprehensive understanding and sets it apart from related concepts [88]. The findings of this study revealed that physical and emotional interaction are important in achieving sexual satisfaction in postmenopausal women. According to Dundon et al.’s (2009) study, sexual satisfaction has two dimensions: emotional and physical [33]. In general, emotional responses include sensations of pleasure, levels of satisfaction or happiness, and pleasant feelings [55]. Relationship happiness, identification, and mutual understanding are the causes and results of sexual satisfaction [35, 39]. In addition, according to Walfisch’s (1984) study, sexual satisfaction in postmenopausal women is influenced by emotional aspects of the relationship and in women, interpersonal relationships and emotional aspects are more influenced by sexual relationships [23]. In explaining these findings, it can be stated that mindful people, particularly during sex, can create harmony between physiological aspects and mental sexual stimulation, and this harmony can have a significant positive effect on women’s sexual experiences [28].

When sexual satisfaction is conceptualized as the sensation of physical gratification, the focus of analysis is primarily on the body and the physical experience. This model places a higher importance on physical and physiological reactions, often highlighting orgasm as the most easily quantifiable indication of sexual satisfaction [33, 65]. Davison et al. (2008) found that having more satisfactory sexual relations increases the number of days of sexual activity and sexual events per month, as well as sexual satisfaction [8]. People who engage in relatively frequent sexual activity report higher levels of sexual satisfaction than those who have less sex [34]. In fact, studies have linked sexual satisfaction to the frequency of sexual relations [39]. In one study, those who were sexually active and satisfied reported higher levels of sexual satisfaction than those who were sexually inactive [30]. The frequency and consistency of sexual activity influence sexual satisfaction during menopause [65], which is consistent with the findings of our study.

The results of this study showed that the fulfillment of expectations has a profound effect on sexual satisfaction. According to Neto (2012), a person’s judgment of sexual satisfaction is formed by comparing her circumstances to the appropriate standards she imagines [98]. Sexual satisfaction in menopause is an assessment of a woman’s level of satisfaction with her sexual life [99]. Philippsohn and Hartmann (2009) define overall sexual satisfaction as a person’s evaluation of sexual life independent of sexual activity [100]. Furthermore, sexual satisfaction can be an interpersonal cost-reward model, considering the amount of cost in the relationship followed by the amount of fulfillment of expectations [33]. McClelland (2010) showed that people’s sexual expectations can vary greatly. When a person’s life is compared to a self-created standard and her conditions match these standards, the person reports high satisfaction [101].

The findings of our research showed that menopausal women deal with behavior and ethics changes, use sex as a tool to express interpersonal relationships, and find sexual satisfaction through emotional fulfillment even in the absence of sexual activity. Non-genital contact and only focusing on the emotional relationship may be important for feeling sexual satisfaction, the perception of intimacy or emotional closeness, and less emphasis on physical acts has a significant effect on sexual satisfaction [8, 35, 36]. They also understand intimacy or emotional closeness and realize that placing less emphasis on physical actions increases sexual satisfaction. Another change in behavior for women during menopause is seeking treatment. When experiencing vaginal dryness, women may engage in various sexual activities aside from vaginal sex or combine therapeutic aids such as lubricants or emotional closeness and place less emphasis on physical actions [45]. The re-emergence of sexual desire in postmenopausal women aids in enhancing sexual satisfaction [11]. Menopausal women aim to lessen menopausal symptoms and enhance menopausal function, according to Niazi’s (2016) [61] review study. Therefore, plants that improve dyspareunia and sexual function can also increase sexual satisfaction (examples include using Tribulus terrestris, fenugreek seeds, fennel, and date pollen) [61], which is consistent with the findings of our study.

The findings of our research showed that the concept of sexual satisfaction is influenced by the change in sexual capacity during menopause. Movahed’s (2019) study found that 35% of postmenopausal women had sex less than once a month, while 45% had sex once or twice a month [63]. Similarly, Merghati-Khoei et al (2014). discovered that libido decreased or disappeared in 94.5% of 200 cases following menopause [102]. Islam et al. (2018) reviewed 34 related articles, including 24,743 Asian women, and concluded that low libido is common in the postmenopausal population [103]. According to women’s reports, menopause can cause various issues that affect their sex lives, such as vaginal dryness, painful intercourse, decreased libido and arousal, and difficulty reaching orgasm [67]. A study on middle-aged Australian women revealed that menopause led to a decrease in arousal, orgasm, and sexual pleasure for all women. Specifically, menopause was associated with increased pain during intercourse, decreased sexual desire and frequency, and less positive feelings towards their sexual partner [34].

Menopause affects women physically, psychologically, and socially. After menopause, women experience a significant decrease in sexual activity and libido, as well as anxiety as a result of their reluctance. Indeed, menopause-related psychological damage has an impact on sexual satisfaction [57]. Middle-aged women may be especially vulnerable to decreased sexual satisfaction due to the increased risk of psychological disorders associated with menopause [33]. Lack of self-confidence caused by a negative body image has a direct relationship with sexual satisfaction and is one of the psychological factors that suffer during menopause [57], which is consistent with the findings of our study.

Sexual satisfaction increases with the rehabilitation program [76] as does mental health improvement [30]. Changing attitudes toward sex, as well as sexual changes associated with aging and menopause, are also significant predictors of sexual satisfaction [8]. Strengthening emotional relationships, increasing intimacy between husband and wife, increasing self-confidence, improving physical and mental health, and raising awareness about the menopause process and the resulting physiological changes will make entering this stage of life more pleasant and lead to greater sexual satisfaction [24], which is consistent with the findings of our study.

The strengths of the present study were the integrated review design in all types of studies and the inclusion of studies without historical restrictions. The limitations of the research were data analysis and interpretation of findings with a subjective approach.

## Conclusion

Antecedents of sexual satisfaction in menopause are a broad self-evaluation of external, internal, and interactive factors. A collection of perceptions, beliefs, and behaviors that are heavily influenced by underlying factors (individual and contextual variables, interpersonal interaction patterns, spouse-related variables, psychological variables, cultural and religious values, and beliefs). In this regard, the concept of sexual satisfaction in menopause is a subjective (emotional interaction) and objective (physical interaction) concept that is conditioned by the fulfilment of expectations and the reconstruction of sexual relations while also being influenced by the change in sexual capacity during menopause. As a result, the couple’s physical health improves, as does their mental health, allowing the marital relationship to continue satisfactorily. This integrated review provided a clear understanding of sexual satisfaction in postmenopausal women, the results of which can be used both in theory and practice. The characteristics of the concept of sexual satisfaction were identified in the research findings. These features in the study it provides a deeper insight into the sexual satisfaction of postmenopausal women. It also helps sex therapists to correctly diagnose sexual dissatisfaction in women. Our scientific findings can be a foundation for future research to increase sexual satisfaction in postmenopausal women. Considering the distinct concept of sexual satisfaction in postmenopausal women, a qualitative study regarding sexual satisfaction in postmenopausal women and the creation of a questionnaire specifically for this age group is suggested.
